# "The Hospital" Nursing Mirror

**Published:** 1897-07-03

**Authors:** 


"The Hospital" Nursing Mirror.

Being the Nursing Section of "The Hospital."

[Contributions for this Section of "The Hospital" should be addressed to the Editor, The Hospital, 28 & 29, Southampton Street, Strand, London, W.C., and should have the word "Nursing" plainly written in left-hand top corner of the envelope.]

### News from the Nursing World.

### UNTRAINED NURSES IN WORKHOUSE INFIRMARIES.

The nursing staff at the City of London Union Infirmary possesses at the present moment too large a percentage of untrained women. Dr. Great-Rex pointed out that 18 out of 37 had little or no experience. It appears, also, that the epileptic ward has, until very lately, been without a charge nurse; and the one recently employed, although she has given much satisfaction, especially to her patients, is to be withdrawn. We trust that the Board of Guardians will very seriously reconsider this intention. Epileptics require the most skilful handling, and if neglected are apt to be dangerous both to themselves and others. The danger of leaving them without trained supervision is increased by the fact that their malady is recurrent at uncertain intervals. Surely it is very false economy to lock the stable door after the steed is stolen; in other words, why wait a tragedy before taking common-sense precautions? We notice that for the future nurses trained in provincial centres are to be considered eligible for positions on the staff. Applications and testimonials may be submitted by post, and if a candidate be considered suitable, and asked to present herself for a personal interview, travelling expenses will be allowed.

### A TRIBUTE TO THE TORQUAY NURSING INSTITUTION.

Not very long ago certain strictures upon the Torquay Nursing Institution were made at a public meeting. It is gratifying to find from the testimony of many Torquay residents that the criticism was quite undeserved, and in a recent issue of the *Devon City Standard* a letter appeared speaking from personal experience in the warmest terms of the nurses and their work.

### RAISING FUNDS FOR SICK CHILDREN.

The fête at the Botanical Gardens in aid of the Victoria Hospital for Children was a delightful affair. It resembled nothing so much as a garden party. The numerous stalls, many bearing the names of ladies celebrated in one way or another, were in tents, and stands dotted about the grounds. The dresses of the ladies of the Pompadour band, the picturesque costumes of the little Morris dancers at the maypole, the medley of handsome and grotesque garments at the stalls and shows, lent interest and variety to the scene.

### NURSES AND THE PLAGUE.

Although we learn that the plague is practically over in Bombay, the spread of the disease to various districts has kept the nurses in various hospitals fully occupied, and some have been transferred from place to place. Nurses Oran, Steele, Franklin, Goulden, Sykes, Briggs, and Mitchell left Bombay recently to take up work at Cutch Mandire. With them went four ayahs, three hospital assistants, and six orderlies for plague duty, beside three servants of the nurses.

### NURSES' HOME IN AFRICA.

In February of 1896 Miss Wild organised the nurses home at Salisbury, Rhodesia. The institution contains only four beds, but 72 sick folk have been treated in it, and out of that number only one died. As usual, people want more of a good thing, and the home is to be enlarged.

### READING'S COMMEMORATION OF THE RECORD REIGN.

Reading celebrates the long reign by founding a nursing institute. Her Majesty has been pleased to approve its being named "Queen Victoria's Institute for Nursing the Sick Poor of Reading, in affiliation with the Queen Victoria's Nursing Institute." The Princess Christian is to be invited to become patroness. The question whether or no artisans should enjoy the privileges of the new institute upon suitable payment is being thoroughly discussed by the subscribers, and is not yet decided.

### BLACKBURN'S NURSES.

The year-old Nursing Association at Blackburn shows healthy growth. In the opinion of its supporters it rivals the infirmary in its usefulness, and contributions flow in freely. But its popularity has created a demand for more nurses on the staff, and consequently the need for yet more liberal support.

### THE CHILDREN'S REJOICINGS AT GREAT ORMOND STREET HOSPITAL.

In commemoration of the "Record Reign," the members of the committee presented the little patients in the hospital on Jubilee Day with cakes, sweetmeats, and dolls, the cost being borne by private subscription. The president, the Duke of Fife, sent each child an enamelled mug, bearing the Queen's arms and portraits of Her Majesty in 1837 and 1897.

### TROUBLES AT BRADFORD.

If there be no smoke without fire, then must the flames be very near the surface at Bradford Workhouse Infirmary. Surely are the ways of guardians, in some cases, past finding out! A grave charge has been made in writing, not by one person, but by half a dozen old and valued servants, against the authorities of that institution, and there is a cowardly or self-interested desire manifested to hush the matter up, and the Nursing Committee have effectually prevented the accusers coming before the board. The facts are these: Six nurses threw up their appointments in a very short period; and Miss Hayes, the last, couched her resignation in the following terms—"It is with deep regret, after four years and six months' faithful service, without, I believe, one single complaint, that through unkindness, injustice, and mismanagement, I tender you my resignation." The charge is substantiated by a "round robin" endorsed by the other nurses who have resigned. These ladies ask to bring their complaint before the Board, but the Nursing Committee accept their resignations on behalf of the Board, and not only throw difficulties in the way, but permit slanders to be circulated against them. Their demand is a perfectly legitimate one, and the refusal to comply with it not only lays the management under a strong suspicion that the grievance is true, but it denies those who make it due reparation for what they have unjustly suffered.

### BAD TASTE AT ABERDEEN.

WE regret to learn that twelve young women, members of the friendly societies of Aberdeen, took part in the demonstrations on Hospital Saturday dressed in the garb of the hospital nurse. There were three collecting carts in the procession, and four women were in each cart. Last year some unseemly displays of this kind were to be seen in some of the processions organised by provincial towns; not only nurses, but patients in their last agony were represented. This wilful harrowing of the feelings is in the worst possible taste, an d must alienate the public sympathy.

### MEDICAL WOMEN.

AMIDST the retrospective criticism which has been brought to bear upon all matters and classes at Jubilee time, that very distinctive product of the reign, the medical woman, has been almost forgotten. We are glad to note that one medical journal has pointed out that in this direction the Victorian era has marked a very important departure in the traditions of the medical profession, and so rapid has the growth of the innovation been that it is difficult to realise that in 1865 only the first diploma was given to a woman, in view of the meeting held at the Royal Free Hospital t is week, when the prizes were distributed to so large a number of earnest women preparing themselves for the medical profession.

### THE QUEEN'S NURSES' COMMEMORATION FUND.

A LETTER signed by the Duke of Westminster, chairman; the Hon. Sydney Holland, hon. treasurer; Harold Boulton, Esq., and Earnest Flower, Esq., hon. secretaries, of the Queen's Commemoration Fund for the benefit of the Jubilee nurses, pleads for increased support. The subscriptions reach £50,000, only half the sum needed to put this institution in a satisfactory financial position.

### A PATIENT'S APPROBATION.

A. ROBERTS, writing to one of the Birmingham local papers, bears grateful testimony to the attention and care bestowed on his case at the General Hospital, and to the kindly interest taken in it by the medical officer and students. This is the more satisfactory as at "Queen's" the attention and interest were conspicuous by their absence.

### DISTRICT NURSES' ASSOCIATION AT MALLOW.

THE second annual report of the above association is pleasant reading. Miss Daly, the nurse, has gone steadily and tactfully about her work, so that there has been no friction, and the association has a satisfactory balance-sheet.

### VOLUNTARY LOCK HOSPITAL, BRISTOL.

THIS institution has been removed from the old premises in Old Park, which were incommodious and inadequate, to new ones in Ashley Road. The record for last year is satisfactory, 37 out of 50 patients being restored to health.

### NURSING LECTURES IN AMERICA.

THERE is one branch of nursing education in which so far, America is in advance of England—that appertaining to lectures and demonstrations. Nurses in England yet need to learn the value of post graduate lectures. In America teachers and nurses are ready, the first to impart, the second to receive necessary and valuable instruction in continuation of their training curriculum. At the Bellevue Graduates' Association of Trained Nurses, for instance, there has been organised a course of lectures and practical demonstrations for the benefit of its members. The first of the series was given recently by Dr. G. M. Swift on typhoid-fever nursing. Following the lecture a demonstration of the bath treatment was given by Miss M. E. Hirschliffe, who also gave an illustration of the dry-pack treatment, with ablution for tonic effect in nervous diseases.

### HARPENDEN CONVALESCENT HOME.

SIR BLUNDELL MAPLE has been royally generous this year, and the convalescent home and almshouses at Harpenden is only one of several handsome gifts. Both are for the use of the employés at the business premises in Tottenham Court Road. The site, a picturesque one, the building replete with every modern convenience and furniture, as well as a substantial endowment, are entirely provided by the donor.

### SHORT ITEMS.

PORTH COTTAGE HOSPITAL has received a cheque for £50 and an annual subscription of £5 from Miss Thomas, of Llwyn Madoc.—Walsall is still divided in opinion as to the respective merits of an eye hospital or a nurses' home as the best commemoration of this year of grace. As far as we can see from the printed matter before us the nurses' home is the more useful.—On Jubilee Day the Lady Mayoress very kindly provided 170 seats in St. Paul's Churchyard for representative nurses from St. Bartholomew's, London, Guy's, University College, Evelina, Victoria, Middlesex, Metropolitan, and King's Cross hospitals, the Hospital for Sick Children, the Hospital for Children and Women, and the East-end Hospital for Children.—The Superintendent of the Trained Nurses' Institute, Weymouth, arranged a little festival on Jubilee Day for her staff. The house and tea-room were prettily decorated, and as many of the medical men belonging to the town as could spare the time were present. The nurses were presented with white sailor hats, trimmed with dark blue band with the name of the institute in gold letters, in honour of the occasion.—Nurse Sarah Hayes, one of the prize-winners in Messrs. Southall's competition, in addition to her training at the Royal Infirmary, Edinburgh, holds a certificate from the City of London Maternity Hospital.—Miss Reckless, of the Winter Street Hospital, Sheffield, has resigned her appointment as matron in order to train as nurse in a London hospital.—The Visiting Committee of the St. Pancras Infirmary invited a number of guests to a general inspection of the institution on Friday, June 18th.—The Committee of the Bristol Royal Infirmary have decided to grant their nurses a week's extra holiday to commemorate the Diamond Jubilee.

### Practical Aspects of a Nurse's Life.

By Sister Grace.

### I.—PROBATIONERS IN THEIR FIRST YEAR.

To be a really good and valuable nurse you must not allow anything else to absorb even a portion of your life. Nursing is an exacting occupation, and to do it well everything else must be given up—society, friends, even home ties. Nothing must enter into competition with your nursing life. It is a grand and noble life, but unless you can give up all to carry out that life, so that having once put your hand to the plough you will not wish to look back, do not enter upon it. Perhaps you think this is a hard saying of mine, but I speak from experience. I advise all to think the matter out thoroughly before embarking on a life which, happy as it is, means a life of entire self-sacrifice. On account of the difficulty of keeping up home ties, I think women who are only daughters or only sisters are not the most suitable to become nurses. The dissatisfied nurses I have seen were those who tried to keep up two lives, one in nursing and one in the world. I think the great happiness I have experienced in my eleven years of hospital life is greatly due to the fact that I resolutely put all else out of my life, and so I had no uneasy longings after the gaieties and socia pleasures of my past life.

We are now to consider probationers in their first year's training. Let us then see what is absolutely essential to success.

### Health.

Primarily, good health. It is mere waste of time, money, and temper to begin nursing without a large reserve force of sound health. It is a hard life, and nothing can alter that fact; outsiders never realise when they visit the wards in the comparative quiet of the afternoon what wear and tear of body and mind have gone to the attainment of those pleasant surroundings in the bright, fresh, spotless wards. A dear old friend came to me one day on behalf of her lady's maid (a poor, discontented, fanciful creature by the way), and this is how she put her request that I would receive this treasure as a probationer: "You know, my dear ——, Clara is so very delicate; she finds the standing and sitting up for me when I am late at balls so very trying, that we think she had better try nursing; it is a nice, light employment." It is needless to say we did not secure this invaluable person for our nursing staff.

Secondly, an even temper is essential; little rubs are sure to come, to say nothing of larger trials, and can only be met and overcome by unfailing good temper.

Thirdly, an ability to efface oneself entirely will help greatly to soften the acerbities of nursing life.

### Outfit.

The outfit is a matter for thought, and not to be overlooked as of no consequence. Probationers have very little time for needlework, so that it is desirable that their clothes should be new if possible, and sufficient to last two or three years without much mending. They should be of light woollen, because nurses become heated in doing unaccustomed work, and then are obliged to stand in draughts, or go into a room of a lower temperature. Shoes must not be considered beneath attention. Always have two pairs in wear, as a change in the middle of the day often proves a great relief to tired feet. Have them of soft French kid, a good fit, and with half heels; absolutely flat shoes are neither comfortable nor good for the feet. Avoid patent leather and prunella.

### A Fresh Appearance.

It is important that a nurse should have a pleasing appearance, bright, neat, and fresh; to that end keep your hair always scrupulously neat. When I began to nurse there was a great prejudice against fringes. I was severely ordered to comb mine down, but when I tried to obey the command the effect was disastrous, as my hair curled frizzily all over my head and was of that tint that friends call auburn and enemies red, and my appearance was that of a startled mediæval saint whose aureole required attention. I therefore compromised matters by arranging the cap, which was of a sensible size, to cover the offending locks as much as possible. This objection seems to have died out now, and so long as you do your hair neatly I think no matron would find fault with the style. Neatness and freshness should be the two words always in your mind with regard to your appearance. Do not be too hurried in the morning to attend to these matters. At first you will find it very difficult to keep clean; some nurses have a natural aptitude for doing a maximum of rough work and acquiring a minimum of dirt in the process. N.B. They generally turn out well.

### Menial Work.

Now let me say a little on the subject of so-called "menial" work. To my mind it is an incorrect term; no work is "menial" that ministers to the comfort of the patients; and if a nurse is ambitious to be one day a ward sister, a thorough and practical knowledge of "menial" work is essential. I am proud to say that I think I could beat most general servants in scrubbing a floor, blacking a grate, or scouring saucepans and tins. A sister should be able to perform all the duties of those who are subordinate to her, and to do that work better; it is a very efficient way of securing their admiring respect. To have this power she must have had a very strict and even a severe training as a probationer. You will frequently hear the opinion expressed that this sort of work is unnecessary for a nurse. Do not believe it. I have shown you its importance for hospital work, and if you mean to take up private nursing it is no less so in that branch, because you will often be so placed, either from the poverty of the patient or other causes, that all this work must be done by you. Nurses who come from the upper classes do this kind of work the most cheerfully. "Noblesse oblige."

Now as to sweeping usually done by the probationers immediately after the beds are made. There is more skill needed in sweeping a room than you would suppose. The first day I set about it I managed to sweep everything round my own ankles, thereby bringing on my head very scathing remarks from some of the patients. Turn up all the quilts and set to work with zeal, tempered by discretion, lest you should overhear some such remark as was addressed to me by an old soldier, who considered himself competent to do everything, from sweeping a room to taking command of the British army. He had gazed upon me for some time in a disapproving manner, solemnly shaking his head, adorned with a tasseled nightcap, and at length the oracle spoke: "That won't never do, miss. Why, you're a-sweeping all that muck up your own clothes. My word, what a mort of washing you'll have to give them stockings." Never be content until your scullery and all the paint that is washed in the wards, such as skirting boards, finger plates, &c., will bear closest inspection. Dusting too is very important, and should be done with a damp duster, otherwise you will only flick the dust from one object to another instead of actually removing it. A sister under whom I first worked, and to whose watchful vigilance over my shortcomings I owe a debt of gratitude, would frequently go round the skirting board with her handkerchief, after the morning's work, and woe be to us if the handkerchief showed a mark.

### Post-Graduate Clinics for Nurses.

By a Trained Nurse.

### XX.—VARIETIES OF REST PATIENTS AND "HABITS."

In the rest-cure wards there may always be found a fair proportion of sufferers from the alcohol habit—women who have long been accustomed to eau de Cologne and methylated spirit indulgence. And it is surprising how much degradation may be comprised in a bottle of eau de Cologne, or a harmless looking bottle of methylated spirit procured for "the heating of curling irons." The men alcoholic patients are mostly the result of innumerable and uncounted small drinks taken at odd times. Another class of patient—and I shall have something to say about each class in turn—are those generally of the more educated type, and frequently medical students or nurses, who have acquired the habit of self-administered hypodermic injections. The hypodermic habit generally carries with it a history of worry, anæmia, and getting below par. The anxiety and sleeplessness often accompanying this condition may at first suggest a hypodermic as a ready means of oblivion, and so on till the patient finds he takes the injection absolutely against his will and better judgment. And he may go on for a long period alternately resisting and yielding, till he becomes a type of that kind of mental and physical wreck which goes to fill the beds of a rest-cure hospital. Perhaps the very worst form of nervous prostration and human wreckage is reached by what are known as the "alcohol jaggers." This is so peculiar a term that I feel an explanation is certainly due to my readers; and I am not at all sure that the condition has any parallel in England. The "alcohol jagger" is a person who takes his alcohol in hypodermic form. He finds that whiskey injected in this fashion has a quicker stimulative result, and that for a smaller quantity of the spirit imbibed he can get a more ready and effective return. He or she, too, runs less risk of discovery, for taken in this form the breath does not cause the individual to stand revealed as a drinker or drunkard.

The "alcohol jaggers," mentioned before as types—among many others—of rest-cure patients, are usually reduced by their constant whiskey or other spirit hypodermics to the most deplorable condition of depression, varied by wild excitement, and marked at all times by an almost uncontrollable craving for indulgence in their terrible custom. Large numbers of patients, too, will be met with who are undergoing the treatment owing to the formation of a habit of hypnotics, which, by long usage, brings them to such an extremity that natural sleep is a practical impossibility. An indulgence in bromide is perhaps the commonest form the hypnotic habit takes in the United States. And the reason of this is not far to seek, for some preparation of bromide is kept as a matter of course in every American home, just as tea and sugar will figure among the household stores. When staying with friends, if one happen to come down to breakfast complaining of headache or malaise, a bottle of bromide in an effervescent form is at once produced, and a dose mixed in the most matter-of-fact way as if bromide were the most innocent beverage, and as mild as a homœopathic globule. And I have repeatedly seen doses of these bromide mixtures—of which there are perhaps fifty different varieties on sale—given to tiny children and babies for restlessness and excitability. Little girls playing about in the sun and feeling hot and headachy as a natural result are given bromo-seltzer, or some such innocuous-sounding a mixture, by their mothers, who seem quite unaware of the terrible results to health of such a practice. Bromide mixtures under various attractive sounding titles, made pleasant to the taste, fizzing and cold, are kept on draught in the chemist's shops. And it is quite a matter of routine for a woman or girl who go out for a morning's shopping or sight-seeing to say, "I feel so tired and nervous; I'll go and get some bromo-seltzer to rest me." And she goes into a chemist's shop, where at the "drinks fountain" she may invest her five or ten cents in most deadly draughts of drugged effervescent waters. From the force of habit it never seems to occur to such girl or woman that she is indulging in the most terrible form of "nipping." Indeed, a large percentage of these women will be total abstainers and express the utmost horror of alcohol in any shape or form. That phrase, "I feel so nervous," is the bane of American existence, and such an expression is constantly coming from "the mouths of babes" and from little girls who ought never to have heard of these a morbid condition. Out in the country parts of the Wild West, where theoretically good health and a complete absence of "nerves" should abound, the same state of things is to be found, and farms, the most remote from the evils of civilisation seem nothing more nor less than manufactories of material for rest-cure hospitals. For even here bromos and bromides and "chloralos" and myriads of other soothing and restful mixtures are hawked round by itinerant quack medicine vendors, who find no difficulty in persuading the tired and restless femininity on these farms that dyspepsia, nerves, and every ill under the sun will be cured by soothing mixtures. I have seen dozens of young women from the dairies and poultry-yards of country farms brought to our rest-cure hospital who have continually dosed themselves for months and years with "soothing mixtures" till they have lost every semblance of human health, and every particle of soundness seemed gone for ever from their original constitutions. This description of a national indulgence in rest mixtures is not one whit exaggerated, and it is set forth as the result of many years experience of life in the United States. A very large proportion of rest-cure patients have their cases complicated by gynæcological disturbances, displacements, retroversions, and ovarian difficulties. And for this, among many other reasons, their nursing is extremely interesting and the field opened up is very varied and calls for experience in many branches of nursing. Sometimes the gynæcological difficulty has produced the nervous prostration—has been the primary cause underlying the tendency to run down and become the nervous flotsam and jetsam and human wrecks most of these patients are. In other cases the nervous exhaustion has so lowered the vital forces as to produce organic anæmia and want of tone. When in charge of a Weir-Mitchell hospital I found the work so extremely interesting that I took careful notes of all the different types of cases, and these notes will enable me to set forth—I hope to the benefit of my readers—the actual steps whereby some of the terrible wrecks of humanity brought to us were restored to some semblance of health. My personal experience of these cases has not, however, brought me into relation with many of the marvellous cures wrought by rest-cure which some persons have been so fortunate as to meet with. At the same time, it is certain that no one who has actually worked in rest-cure wards, and has seen the practical results obtained, can fail to be impressed with the wonderful value of perfect quiet, inaction, and judicious feeding. But I am sorry to say I have never had the personal care nor shared in the triumph of helping those human skeletons of whom we hear, persons haunted with aches, pains, and enduring terrible sufferings to emerge after about six weeks of massage and forced feeding as rosy, plump, beautiful women, with every organ rebuilt and renewed, and no trace left of nerve depression and exhaustion. In fact, I have not myself seen the total banishment by the miracle of rest of every ill that flesh is heir to. I have, however, seen good practical results and enormous benefits accruing to those patients who came under treatment during the earlier stages of their physical degeneracy.

### A Nurse's Visit to Texas.

V.

Everyone rode in Texas, and my brother set apart a pony for my special use. Twice a week regularly, to his great disgust, I fetched him up from the pen, saddled and bridled him, and rode to fetch the letters from the little store three miles away, where the mail man left them on his way from Austin to Fredricksburg. Sometimes we would go either in the wagon or on horseback to spend the day with some of our neighbours, and twice during my seven months' visit we had fishing picnics for change of air and holiday. These were grand occasions, we took our bedding and provisions for three or four days, and drove about ten miles to a large river, the Perdernales, where there was good fishing to be had. I suppose we were not expert, for we only caught small fish, and not many of those, so that, as we had depended largely on sport to replenish our larder, we found one morning, on examining our stores, that all we had left was flour, tea, sugar, pepper, and salt. Next picnic we were more provident.

We spent our days, the others principally in fishing, and I in exploring. Everything was new to me; fresh birds, insects, trees, and flowers everywhere, and some parts seemed a kind of fairy land, especially where I first saw humming-birds at home. I found them through their humming; they were not the brilliant living gems of the tropics, but dainty, sober little fellows in grey, with green heads.

Camping out was delightful in warm, dry weather. The wagon tilt was put up, Clara and I made up our beds inside, while Henry slept on the ground underneath, with "Don," the dog, beside him to keep off intruders in the shape of inquisitive cattle, which often came round the camp at night to chew the corners of the wagon-sheet, rope-ends, or anything in the way of clothes they could find left about. I was told they found a certain amount of saltness in these things, and that was why they did it.

The first night I undressed and went to bed properly, but afterwards found it much better to sleep in my clothes ready for anything that might turn up, such as a horse breaking away and having to be caught, or a fierce norther, *i.e.*, north wind rising suddenly.

It was great fun having one's morning wash in the rushing water among the big stones on the river bank; far pleasanter than in a little basin perched on the wagon front.

It gave one such a strange solemn feeling sitting by the camp-fire at night, just that little patch of light with darkness all round, the sound of the wind in the trees, the river rustling among the rocks in the valley below, with wolves howling in the distance; while now and then, just outside the circle of light, footsteps and rustling could be heard in the brush—only cows; but twenty years earlier Indians frequented this river, and then it was no place for pleasure picnics. Those were stirring times for the settlers. The older ones used to tell me tales of anxious nights and false alarms till they made me feel creepy. The Indians chose the time of the full-moon for their raids, so at that time every month the rancher brought his horses up to the house at night, and slept outside among them, with his fierce dogs to give the alarm, and his loaded gun ready to his hand.

In those days all women learnt to ride barebacked, that if they were unfortunate enough to be taken prisoners by Indians they might have a chance of escape; and even now it is not uncommon to find Texan girls who can canter over the country, carrying a smaller child in front of them, and with only a sack on the horse's back. Now the Indians are shut up in their reservation, the Indian territory to the north-east of Texas, the interloper can sleep in safety on the old hunting grounds, and the only visitor the lonely ranch is likely to have after nightfall is some traveller on foot or horseback asking for a lodging, which is never refused him out there.

### News from Greece.

From a Correspondent.

Since I last wrote we have been honoured with a visit from the Queen and Crown Princess. They came last Saturday and stayed nearly two hours, visiting each patient and inspecting all our hospital arrangements, with which they expressed themselves very much pleased. We have discharged several minor cases and taken in others. Our cases are now becoming more medical than surgical, and the medical are the most severe. Amongst the wounds the worst have been those of the chest and lungs. I have now a very bad case in my wards. The bullet entered near the base of the sternum and passed out under the right arm, leaving something in the wound, probably either cloth or flannel from the clothing. The patient is too ill to stand an operation at present. Probably if his strength be maintained a portion of rib will be removed, but he has had a bad diphtheritic throat, and his temperature was 105 degrees for a day or two, so his case is serious. However, we hope he may pull through, as he shows signs of improvement. The Queen was especially interested in his case, her kindness quite overcame him, and his temperature rose with the excitement. Our other cases are all doing well.

The midday heat is terrific, and we feel it greatly. It interferes with our rest at night, and we are nearly bitten to death by many and various kinds of insects, which abound everywhere, and which seem to delight in English blood.

If we decide to undertake the medical cases, as they wish us to, we shall be here a long time; if not our work, I think, will soon be done. Mr. Davis and Mr. Fox-Symons are also here, and Mr. Abbott, who is visiting Athens, intends to return.

As the steamers land some of the sick here, we occasionally received hurried orders to prepare for thirty patients—a large number after the two or three a day which we were accustomed to regard as a good day's work at home. The patients' clothing is a trial, as it is infested with vermin; we fold it in bundles, label it, and consign it to an outhouse until wanted.

We have an army of refugees; 9,000 have bread given them daily, and 5,000 receive help. They lie sleeping about everywhere—in the streets, on the cathedral steps, and in the square. The children seem to enjoy it, looking on it as a continual picnic, but their elders suffer, and it is well for all that they live in a warm climate.

When I write next I will tell you about the prison here, which is very old, and in some respects very terrible. I am sorry not to send you better letters, but time is money here.

### Ormskirk's Intentions.

The opportunity is considered favourable for adding accommodation for the housing of the district nurses to the Cottage Hospital. This can be done at very little additional expense. It is proposed to provide for six nurses, who will be employed amongst the sick within the district covered by the Ormskirk Dispensary, *i.e.*, six miles in any direction from the market place of that town. The services of these nurses will be free to the poor, but the better off will be expected to contribute to the funds of the charity. It is also intended to make arrangements whereby private nursing can be undertaken, and thus bring the advantages of this nursing centre to all classes. The hon. secretary is appealing for an endowment fund for the new home, and proposes to name it "The Queen Victoria Record Reign Memorial Fund."

### Talks about Food.

### GILES AND HIS FOOD MONEY.

Not long ago a writer in one of the magazines gave us the weekly *menu* of a country labourer's family, taken down from the lips of his wife—a *menu* which strikingly bears out what we have said as to the monotony and lack of resource in the matter of diet displayed by the poor. As a general rule she had 9s. 3d. available for food; and of this 8s. 4½d. went in materials for bread, the remaining 10½d. being spent as follows: Butter, 3d.; sugar, 4½d.; and tea, 3d. Think of the deadly monotony of such a diet, even when supplemented, as no doubt it was, by the produce of her little garden! Bread for breakfast, bread for dinner, bread for tea; butter, sugar, and tea being generally more conspicuous by their absence than by their presence—for how long could they last with a party of ten? Perhaps a "light pudding" may now and then have varied the bill of fare; but no, even that could not have been made without milk or eggs, and of these articles, except as a very occasional thing, there were none. Yet even this meagre bill of fare is beaten by one known to the present writer—that of a family who lived throughout a hard winter on bread and sugar—that almost black sugar which can be bought for the modest price of three-halfpence per pound.

One's first thought on hearing of such cases is, "What terrible poverty!" But in many minds a second thought will follow, and that second thought is, "What terrible lack of resource!" And, perhaps, this lack of resource is more widespread than the poverty. One is glad to know that there are few counties in England where wages will not permit of something more being spent on food than could be spared in the instances given; but when we come to ask whether food money, be it much or little, is anywhere spent as profitably as it might be, we can only answer that in England we can point out no such place. Still, there is no doubt that it is the very poor who are most deficient in enterprise and resource with regard to food—as is only natural, for low living is not conducive to brightness of brain. It is the very poor, then, whom we should chiefly strive to help in this matter; and, seeing that it is of the highest importance to them to spend their scanty wages well, we may do them the greatest service by suggesting variations on the usual bread diet—oatmeal, cocoa, rice and suet puddings, soup, haricot beans, &c., &c. We hazard the guess that greater variety in their food would not make the healthy members of the family cost more to keep, and that very likely they at present eat more in bulk than they really want, in the instinctive effort to provide themselves with certain necessary elements in which white bread is deficient; while as to the delicate ones, a more varied and appetising diet might indeed be more freely eaten, but would probably save doctors' bills, and bring renewed life to many a listless and dyspeptic invalid. It has been well said that "the stomach needs surprising now and then," and especially does this apply to the delicate stomach.

Sickly and delicate people come off terribly poorly with regard to diet, if they happen to belong to the poorer classes. The little sick-room "messes," made by themselves or their friends, are messes indeed; and the ordinary diet of the family needs the sauce of hunger to be palatable—a sauce which they can seldom procure. For one thing, when cooking utensils are scarce, everything seems to taste of everything else, to the marked injury of its own particular flavour. A typical cottage supper, when the cottagers are fairly well-to-do, is that cooked in the "boiler" hung by a chain from the old-fashioned chimney. All the materials for the supper simmer away together, the potatoes in their own net, the onions in theirs, the dumplings in a third, and so on, while the meat (if there be a bit of meat) wanders round at its own sweet will. When supper time comes, the various nets are fished out, and their contents deposited in dishes; and an excellent supper is provided—only, as we have said, everything tastes of everything else, and the fastidious stomach of a delicate person will often turn against such food. It is a great boon to the sickly poor to have simple lectures given by their friends on sick-room cookery, and on the kinds of food which delicate people should take. For lack of knowledge on this latter point, many women with delicate appetites and defective digestive powers drop one article of food after another, and finally take to living entirely upon bread and butter and tea—very little of the former and large quantities of the latter, a diet which can never properly nourish them, and only increases the difficulty of digestion under which they already labour.

But there is another way in which those who wish to see an improvement in the diet of the poor may help. In country villages the system of marketing adopted by the poor is a thoroughly bad one, as is plainly shown by the high prices, or else the exceeding badness—sometimes, we are afraid, it is both together—of groceries at village shops. It is, of course, inevitable that people who have no conveniences for storing should buy small quantities at a time, which doubtless accounts to some extent for high prices, but we believe that the main reason for these high prices is the habit of running into debt. Where bad shopmen are concerned, it is manifest that by getting into debt with them the villagers lose all freedom of action, and are obliged to put up with high prices and inferior goods; and where a good shopman is concerned, the very kind-heartedness which prevents him from pressing for payment, and eventually saddles him (as we know for a fact that it does) with heavy losses, really presses very hardly upon the better class of customers, obliging him to put a high price on his goods, and thus make up, at least to some extent, for his bad debts. Anyone who could persuade such a shopman to encourage ready-money payments would be doing a good deed. By laying their two heads together they might easily devise some system of cash and credit prices, or of coupons for payments over the counter, which would help the poor to help themselves in this matter. Or, if the co-operation of the shopman cannot be obtained, why should not a small "store" be started, where prices should be low, and only ready-money business done?

Here would be a chance, too, for introducing wholesome but little known articles, such as cocoa, oatmeal, haricot beans, &c., and care would also be taken that all articles supplied should be good of their kind—that tea, for instance, should be tea, not sloe leaves and iron filings. Connection with a good wholesale place would ensure this, and enable one also, we feel sure, to sell at fairly low prices. It is hardly to be expected that goods can be cheap which are brought into the little village shop, not by a wholesale man, nor even by a large tradesman, but from a trumpery little town in the neighbourhood. Yet this is the way in which a village "business" is often—perhaps generally—conducted.

### Appointments.

### MATRONS.

Clatterbridge, Cheshire.—Miss Lilian Rice was appointed Matron of the Fever Hospital on June 25th. She was trained at the Adelaide Hospital, Dublin, for three years, and afterwards had eight years' experience as district nurse at Carnarvon, when she became matron of the Bangor Isolation Hospital.

### Sketches of Hospital Life.

### "KNOBS."

About a hundred yards from the hospital a thoroughfare crossed the main street, and here Knobs used to wield his brush. He was a stunted lad of fourteen with a bulging forehead which gained him his name. Of weak intellect, he still had much of the sharpness of the street arab. We patronised his crossing, which led to the station, and he was always proud of piloting a "nuss" across the crowded roadway. Many a penny from our scanty pocket money found its way into the canvas bag which served him as purse and larder. One day I found him in the casualty room with a scalp wound. "Mammy done it with a pint mug" was his explanation. While waiting for the doctor Knobs waxed confidential. I learned that his mother was a certain Mrs. Browne, well known to us and the police. It was evident from what Knobs said that he had a great opinion of his mother. The regard of a child for its parent is only too easily killed by harsh treatment, but his affection seemed to thrive on systematic cruelty. From the time of his "accident" he considered me a friend, and we used to have little chats as I stood at his crossing waiting for a likely gap in the procession of vehicles. About a year afterwards, as I was leaving for a fortnight's holiday, I met the ambulance stretcher in the hall. On it was Mrs. Browne. She had been run over in D—— Street while intoxicated.

On returning, I heard that Mrs. Browne had died shortly after admission. When next at the crossing I spoke kindly to Knobs about her. "She's not dead," he replied quietly. "Not dead!" I cried; "why, they said at the hospital——" "I don't care wot they says," he interrupted, doggedly; "she's not dead. It's this way," he continued hurriedly, as if anxious to check any congratulations, "I was standin' here w'en up comes Joey Tomkins an' says, 'Your mammy's took to the 'orspital. She's a deader,' says he. So I ran along the road, an', sure enough, there was a crowd at the gate. I shoved in an' a gen'leman wot was standin' by the door says, 'Well, my little man, wot is it?' 'It's Knobs,' says a boy in the crowd; 'she's his mother.' So they made me sit on a bench, an' after a bit the same gen'leman come back an' says, 'Your mother's very bad in there, my boy, an' wants to see you.' I went into a big room full of beds. It was dark, an' I couldn't see very well. I saw a woman in a bed that wasn't mother. Her head was all tied up and her face wasn't the right colour. 'It's not her,' says I. 'Hush!' says the gen'leman, 'go up to her. Take her hand.' So I went close to the bed, but the woman on'y groaned and said nothin'. At last the man said I best come away. Next thing I hears she's dead and buried. But I know better. She's gone to M——, an' been took up for drinkin' like she was last year. I expect I'll see her comin' down the cellar steps one of these days."

Knobs searched my face with a furtive fear in his eyes, and I tried to look convinced.

### A CASUALTY.

Two miles westward the Dock Road, on which our hospital was situated, ended abruptly, for beyond there was nothing but waste land bordering the mouth of the river. Here the beach—euphemistically called The Sands—consisted of mud, rough stones, broken bottles, and other *débris*. On Saturdays and Sundays the inhabitants of the slums used to sally forth in hundreds to make merry on The Sands, paddling about in the ooze and splashing in the brackish waters of the river until sundown. These "outings" usually brought us a harvest of casualties. Once, when a thunderstorm, followed by torrents of rain, had ended a stifling day, I was tidying up the casualty-room after the admission of a case when I fancied I heard a child crying just outside the window. It was only for a moment, for the storm clashed with the rattle of traffic, the shuffling of feet on the greasy pavement, the hoarse shouts, shrill cries, and bacchanal laughter of a Saturday night mob in the great Dock Road. My mind, however, was uneasy, and, calling the porter, I told him to look outside. A moment later, I heard him asking questions and the answers coming back in a shrill treble. I gathered that the handle of the casualty bell was working stiffly, and the owner of the voice had been unable to make it ring. Then the porter appeared at the door of the casualty-room. "A cut-foot, nurse," he said, in a hoarse but sympathetic whisper; "a little boy. Two nippers no bigger than my Sammy has carried him all the way from The Sands—two miles on such a night, and along the Dock Road!" Then he stood aside, and three dripping urchins about 10 years of age entered in single file. The last and youngest was limping, and at once made for the patients' chair, on the edge of which he seated himself stiffly. Untying a rag from one foot I found a considerable wound on the sole. Just then the house-surgeon entered. "A nasty gash," he said, "how did you get it, my little man?" "A bit of glass done it on The Sands," sobbed the child. "On The Sands! And how did you get here?" "Billy and Mac carried me turn about." "It's a long way," said the doctor, gravely, "where do you live?" "First house in D—— Street," eagerly replied one of the others, a red-headed boy with plain but pleasant features; "we can carry 'im 'ome in two minutes, quite easy. Me an' Mac promised 'im you wouldn't keep 'im in." "Well," said the house-surgeon, when the dressing was finished, "I'll let him go as he lives almost next door; but remember he must be brought here on Monday morning at half-past nine sharp, and in the meantime must not put his foot to the ground." He slipped something into Billy's hand, while Mac stooped down and the patient scrambled on his back.

The doctor and I followed the trio to the door, where they paused as the rain blew in upon them. The patient's bandaged foot protruded stiffly from under one arm of his friend Mac. Billy evidently felt he should be doing something. He glanced into the darkening night, considered for a moment the snowy bandages which must soon be soaked with rain, then, taking off his cap, placed it on the injured foot, and darted bareheaded into the storm!

### Presentations.

The House Committee of the Clayton Hospital, Wakefield, have commemorated the record reign by presenting each member of the nursing staff and each servant with a memento. The matron received a travelling clock, each sister a gold ring with the hospital's initials and the dates of the Queen's reign, each nurse a golden brooch with dates, &c., servants a silver brooch, porters a silver pendant. The patients were entertained to tea and a concert afterwards by the Mayor.

Sunderland Union Infirmary.—Miss Cowan, who has filled the position of Lady Superintendent of the Sunderland Union Infirmary for over four years and is now leaving to take up a similar position at King's Norton, was presented by the medical superintendent, on behalf of the medical and nursing staff, with a silver cake basket as a token of their high esteem and regard. Miss Cowan is carrying every good wish from those with whom she has worked so harmoniously and successfully.

Wakefield.—Miss Reed, the Principal of the Trained Nurses' Home, presented each of her staff of twenty nurses with a Prince of Wales's Hospital Stamp, neatly mounted on a handsome card specially prepared in two colours in commemoration of the Queen's Record Reign.

### Everybody's Opinion.

[Correspondence on all subjects is invited, but we cannot in any way be responsible for the opinions expressed by our correspondents. No communication can be entertained if the name and address of the correspondent is not given, or unless one side of the paper only is written on.]

### MALE NURSES.

"C. J." writes: It is with great pleasure I read the article referring to male nurses in this week's issue of THE HOSPITAL. Having been continually employed in private work since the completion of my training at the National Hospital, Queen Square, I do not speak without experience when I say there is a great and an increasing demand for male nurses. It has always been a matter of wonder to me why our general hospitals do not open their doors to male probationers. There may often be found cases in their wards totally unfit to be nursed by their own staff. The only remedy the matron seems to have is to send for the only male nurse perhaps available, with probably an outlay of three or four guineas weekly to be faced, and with only a very vague idea as to where her hired nurse has been trained, or if, indeed, he has had any real training at all.

### FEVER NURSING.

"PROVINCIAL MATRON" writes: I have read with much interest the remarks of "Anonymous" regarding fever work. In my opinion, however, no nurse, even with the very best general training, is competent to take charge of fever patients without being properly taught the work. During many years' experience in small fever hospitals where the aid of private nurses was often needed, I have been shocked to find well-trained general nurses, holding first-class certificates, with little or no knowledge of the nursing and management of scarlet, diphtheria, aye, and even typhoid patients, and I have thought what a terrible mistake it was on the part of those responsible to send such to nurse private fever cases where there would be no matron or sister to direct them. No doubt fever nursing is very trying to many; it necessitates a somewhat isolated life, it is impossible whilst engaged in it to mingle so freely in society or to receive visitors as in a general hospital; but, for my own part, I like it, and would not change it for any other. Doubtless it is wiser to become proficient in one thing, but nowadays one cannot (and very properly so) obtain any responsible position in fever or any other branch of nursing without a general training.

### BADGES AND REGISTRATION FOR TRAINED NURSES.

"TRAINED NURSE" writes: It has occurred to me that the following plan would answer if carried out to make the distinction between trained and untrained nurses, viz., that every trained nurse wear a badge marked "Trained Nurse," that each nurse be licensed and only able to procure a badge on producing her certificate; and for anyone wearing the badge to know that any time they may be called upon to produce their licence.

*** This suggestion hardly improves the position of affairs, as it would only offer the public an unreliable guide, for imitation badges can easily be obtained for the work undertaken by nursing associations of repute. The origin and character of the badge would require as strict an investigation as the training and character of the nurse who wore it, and why wear it? The licensing of so large a number of women would be a difficult and expensive matter, and it is impossible to compel people to be nursed by a trained woman if they prefer another.—ED. *T. H.*

### A Matron's Treat.

GREAT YARMOUTH.—Miss Hadway, matron of the Isolation Hospital, gave a pleasant evening to the patients and staff in honour of the Queen's Record Reign. Music and dancing formed part of the entertainment, which passed off most enjoyably.

### Midwifery, Medical, Surgical, and Other Nurses.

### DR. RENTOUL'S LITTLE HODGE-PODGE.

DR. RENTOUL'S energies are absolutely irrepressible. He has now engaged in the hopeless task of trying to "please all parties," and with this object he has decided to extend the scope of his Bill for the Registration of Midwifery Nurses "so as to include not only midwifery, but also medical, surgical, and other nurses." He hopes that this will please all parties, and that we shall soon get rid of an important subject! He is clearly in a hopeful humour if he thinks that the inclusion of all sorts of nurses will simplify the passage of his or indeed of any Bill.

In his former Bill the rights of existing midwives had to be protected by the insertion of a clause enabling them to register. How then about the enormous multitude of women who are now in one form and another earning their living as "nurses." Are they all to be registered? If, as we are told is the case, Parliament is unlikely even to interpose any restriction upon one woman helping another in her confinement, however unqualified she may be, we find it difficult to believe that it will ever consent to interpose restrictions in the way of women officiating as "nurses" whatever offices that term may include.

The difficulties of legislating for the regulation of nurses are far greater than some people think. At the present time there is a general consensus of opinion among trained nurses as to the position occupied by the various training schools, and the qualifications of a nurse are to some extent expressed by a statement of the school where she was trained. But to give a legal definition of the word "nurse" is a very different affair.

Nurses for the sick are quite distinct from nurses for children; some trained nurses scout the idea of being associated with mental nurses, although the latter maintain that they also are trained—there is no generally recognised standard of the training which is necessary; gynæcological and surgical nurses look upon the fever nurses as quite outside the pale; while all the sisterhood of trained nurses regard the monthly nurse as quite inadmissible to their ranks; the poor wet nurse is a pariah; and as for the midwife, on whose account the whole pother has arisen, she not only is not a nurse, but she rather protests against being asked to do the nursing. We see no single element of unity in all this. Sometime or other such an element may arise. Sometime, when nurses have an easy means at their disposal of discovering the training which each of them has undergone, it will probably be found that certain groups—nurses who have undergone training of a certain standard—will be willing to work together and to recognise each other, and it may then be possible to give legislative protection to some distinctive name which they may adopt. In the meantime we must wait, and we may be sure that no attempt to group together midwives and nurses is likely to hasten matters.

### Where to Go.

ON Saturday, July 10th, a musical conversazione will be given by the National Hospital for the Paralysed and Epileptic at Queen's Square, Bloomsbury. Arrangements have been made by Mr. T. S. Ware for a grand show of herbaceous and other flowers, by Messrs. Paul and Son for an exhibition of Royal roses, and by Messrs. Cutbush for a show of carnations. Music will be provided, and the Bijou Band, conducted by Mr. T. Pougher, will perform in the garden. Tea and coffee will be provided. The doors will open at three p.m., and visitors are requested not to be later than half-past three in entering the music room.

### Prize Giving at the Royal Free Hospital.

Mrs. Creighton, wife of the Bishop of London, gave the certificates and prizes to the students of the London School of Medicine for Women at the Royal Free Hospital on Tuesday, June 29th, at half-past four p.m.

The opening statement was read by the Dean, Mrs. Garrett Anderson, who said that 62 new students had joined the school since October of last year, thus placing it third amongst the medical schools for the number of new entries.

This year marks a new development of the work. The school has become a corporate body, and is more closely associated with the hospital. Its name under its charter is the "London (Royal Free Hospital) School of Medicine for Women," and three members of the School Committee take their places upon the governing board of the hospital.

The first portion of the new buildings will be commenced in the garden of the present school. It will consist of laboratories, which will occupy three floors, and it is intended that they shall be second to none in completeness. It will be finished, it is hoped, in October, 1897. The money is in hand to defray the expense. The next block will be lecture theatres and biology rooms, and the third portion will be the residence for students in Hunter Street. It is estimated that seven or eight years will be needed to carry out the scheme if enough money be subscribed. The school was entirely self-supporting last year, there being a balance to the good of £300. This, and a possibly larger one this year, will be absorbed by the new building operations.

Mrs. Creighton's address was admirable. She reminded the students that in the full useful life before them they possessed two gifts in a much greater measure than their sister women—liberty and knowledge. These gifts were responsibilities, and the time spent in the school was a test of their ability to use them in a life of service. To do this worthily they must bring a spirit of reverence into their work, especially reverence for the human body as being the home of the spirit. There was a great danger of students forgetting this spirit of reverence and ignoring the infinite mystery that unites spirit and body. At one time the advance of science made such strides that it became common to imagine that man could acquire all knowledge, but now a humbler spirit was abroad, and it was well to remember however much we might know it was only an infinitesimal fraction of the whole. Now that so many professions that have been man's were being thrown open to women, it was generally found that women did not work in them in a man's way but in a woman's way, and did things that men ignore. It did not yet appear what women would do in medicine. But if they were women first, then they would find a way to serve mankind as men could not. There were possibilities of this already foreshadowed in work amongst the specially suffering, perhaps specially sinful of their own sex, and women understanding the suffering might be able to heal both body and spirit. She exhorted them to be students always. Women had not as yet added much to the sum of human knowledge; they had not the reputation for being careful and plodding workers. Let them be sure, however, that any good, true work done by them would meet with full recognition by men, and was there a greater blessing than to add something to the general welfare? The speaker concluded with an emphatic reminder that neither knowledge nor diligence availed aught for good without the weight of personal character, pointing out that they were as yet pioneers in a new career following noble examples, and it behoved them to set such themselves.

The Chairman of the Hospital Committee proposed a vote of thanks to Mrs. Creighton, which was seconded by Dr. Elizabeth Blackwell.

### The Book World for Women and Nurses.

[We invite Correspondence, Criticism, Enquiries, and Notes on Books likely to interest Women and Nurses. Address, Editor, The Hospital (Nurses' Book World), 28 & 29, Southampton Street, Strand, London, W.C.]

Everybody's Guide to Photography. (London: Saxon and Co. 159 pp. Price 6d.)

This little manual contains much useful information in a small compass. Not only will the beginner find simple directions to guide him in the field and in the dark-room, but details of many processes are given which will interest those who have made some progress in the "black art." It also contains numerous illustrations and a collection of useful formulæ.

"Not By Might." (The Church of England Zenana Missionary Society.)

To those interested in the Zenana work, a feature of latter-day civilisation in India, we would commend to their attention a little book just published dealing with this subject. We allude to the latest publication of the Church of England Zenana Missionary Society, entitled "Not by Might." Here, in one-volume form, we have a briefly epitomised account of the experiences of one who has laboured in this particular field of missionary work. The authoress, who signs herself by her initials only, gives her testimony from the standpoint of an eye-witness to the various scenes described, and her account thus given first-hand is interesting and instructive. That the Zenana work is not free from arduous labours and keen anxiety is pointed out pungently; the path is beset with difficulties. Not least among these in the writer's case were insuperable obstacles in progress of her nursing through want of conversance with the languages of the race she was thrown with in the progress of this work.

### Minor Appointments.

Stockport Union Infirmary.—Nurse E. Ball has been appointed Charge Night Nurse at Stockport Union on June 21st. Nurse Ball was trained at Brownlow Hill Workhouse Infirmary, Liverpool, and has since worked as district midwifery nurse, Merthyr Tydvil Hospital.—Nurse Jessie Peck was appointed on June 21st Charge Day Nurse at Stockport Union. She was previously charge nurse at Gore Farm Fever Hospital, and later matron of St. David's Home, Broadstairs, and was trained at the Hospital for Women, Soho Square, W.

Cutch Mandrie, India.—Miss G. Franklin, of St. Thomas's Hospital, has been appointed Sister-in-Charge of the two Mahommedan Hospitals at Cutch Mandrie, able assistance being rendered her by Sister Oram and Sister Steele of St. Bartholomew's. But a tithe of the actual hardships can be realised by those who do not know the place and people, and that these ladies under these circumstances should voluntarily undertake the charge of a second hospital calls for the highest praise.

Fir Vale Infirmary.—Miss Minnie S. Pruett, who is now acting as Night superintendent of nurses at Fir Vale Infirmary, Sheffield, has been appointed Day Superintendent of nurses at Sunderland Union Infirmary. Miss Pruett possesses excellent testimonials, and her departure will be much regretted by friends, nurses, and patients.

Blackburn.—Miss Elizabeth Dunwoody was appointed Superintendent Nurse at the union on June 26th. She was trained at Brownlow Hill Infirmary in 1882.

### For Reading to the Sick.

PERFECT LOVE.

"THERE is no fear in love; but perfect love casteth out fear, because fear hath torment. He that feareth is not made perfect in love."—1 *St. John*, iv. 18.

**Verses.**

And if some things I do not ask
In my cup of blessing be,
I would have my spirit filled the more
With grateful love to Thee:
And careful, less to serve Thee much,
Than to please Thee perfectly.
—*A. Waring.*

Love is the root of creation,—God's essence!
Worlds without number
Lie in his bosom like children! He made them for this purpose only—
Only to love and be loved again! He breathed forth His spirit
Into the slumbering dust, and upright standing, it laid its
Hand on its heart and felt it was warm with a flame out of heaven.
Quench, oh quench not that flame! it is the breath of your being!
Love is Life, but hatred is Death!
—*Longfellow.*

O blessedness all bliss alone
When thy pure fires prevail!
Love only teaches what is Love;
All other lessons fail.
We learn its name, but not its powers—
Experience only makes it ours. —*Cowper.*

Stronger than steel
Is the sword of the Spirit!
Swifter than arrows
The light of the truth is!
Greater than anger
Is love, and subdueth!

The dawn is not distant,
Nor is the night starless—
Love is eternal!
God is still God, and
His faith shall not fail us!
Christ is eternal. —*Longfellow.*

Blest are the men of peaceful life,
Who quench the coals of glowing strife;
They shall be called the heirs of bliss,
The sons of God, the God of peace.
—*Isaac Watts.*

**Reading.**

"Beloved, let us love one another; for Love is of God, and every one that loveth is born of God and knoweth God."

Verily love either takes away the bitterness of trial, or else gives us courage to accept. It is said that the fish of a certain river shine like gold so long as they are in their own waters, but take them thence, and they become like common fish. Just so with afflictions; if we lose sight of God's Will, they press upon us with all their inherent bitterness, but he who looks at them beneath the light of God's good pleasure sees them glowing, gilt, and precious.—*St. Francis de Sales.*

The love of the World takes away from men a desire after and relish for heavenly things. None of the bidden guests were kept away by any occupation in itself sinful, while yet all became sinful because allowed to interfere with higher objects, because the first place, instead of a place merely subordinate, is given to them.—*Archbishop Trench.*

### Notes and Queries.

**The contents of the Editor's Letter-box have now reached such unwieldy proportions that it has become necessary to establish a hard and fast rule regarding Answers to Correspondents. In future, all questions requiring replies will continue to be answered in this column without any fee. If an answer is required by letter, a fee of half-a-crown must be enclosed with the note containing the enquiry. We are always pleased to help our numerous correspondents to the fullest extent, and we can trust them to sympathise in the overwhelming amount of writing which makes the new rules a necessity. Every communication must be accompanied by the writer's name and address, otherwise it will receive no attention.**

*Bed Pulley.*

(309) Can you tell me of any appliance for adjusting a pulley to a bedstead without using the ceiling? Something a district nurse could carry and fix in a cottage.—*E. G. B.*

The most convenient plan is to fasten a piece of round towelling or broad bit of webbing or rope, knotted together at its two ends, round the railing or footboard at the bottom of the bed, making it long enough for the broad loop to be within easy reach of the patient's hands. Any invalid furniture maker, or bedstead manufacturer, will make an iron upright curving over the head of the bed. It can be made to clamp on to the bedstead, and is easily removed. We hope shortly to be able to give full description, with details as regards price.

*Training at a Children's Hospital.*

(310) I want to be an army nurse, and as I am too young for a general hospital I am going to a children's hospital for three years. Will this training be of any service to me?

Only in so far as all experience is valuable to a nurse. Army nurses must serve three years in a general hospital. Three years is longer than necessary to acquire the knowledge to be obtained in a children's hospital.

*Nursing at the Cape.*

(311) Will you kindly tell me to whom to apply respecting nursing at the Cape under the Victoria Nursing Institute? Also how to become one of Lady Roberts' Nurses?

Write to the Secretary, Queen Victoria's Jubilee Institution for Nurses, St. Katherine's Royal Hospital, Regent's Park, N.W., for information respecting work at the Cape. Lady Roberts receives no applications from nurses. She chooses her own.

*A Bicycle Fraud.*

(312) Please advise me. I bought a bicycle secondhand with no name upon it. It was represented as a "Granville," in good condition, with new tyres. It is not a "Granville," and one tyre is old. Could I employ a lawyer to recover the money?

Certainly; but it would cost as much as the bicycle was worth to do so. Better deal in future with respectable tradefolk, who may charge more, but who for the sake of their reputation will not make false representations.

*Private Nursing on the Continent.*

(313) Can you give me any information respecting private nursing abroad?

"How to Become a Nurse," published by the Scientific Press, gives the names and scope of the most reliable nursing associations, and they generally have a connection with Continental societies.

*Should Nurses Salute Each Other.*

(314) May I suggest that nurses salute one another when they pass in the street. They are bound together by one common bond, in fact, are one great sisterhood, and surely sisters ought to recognise one another.

Is it necessary or even possible that this should be? All women are one sisterhood, a fact which is recognized at once in moments of peril or distress, but this is not obtruded on public attention until need requires it.

*A Book on Drugs.*

(315) Can you recommend a book on the sources, preparations, and uses of various drugs, giving the price of the publication?—*Staffordshire Knot.*

"Recent Materia Medica: Notes on their Origin and Therapeutics, 1891," by F. H. Lescher, F.C.S., 2s. 6d.; and "Organic Materia Medica: Treating of some of the more important of the Animal and Vegetable Drugs made use of in Medicine, including the whole of those in the B.P.," by W. Southall, F.L.S., 8vo., 6s., 1896, may meet your requirements.

*A Question of Maintenance.*

(279) *District Superintendent* asked in the number of June 5th what was a fair and comfortable allowance per week for boarding nurses in district home?

A Matron kindly replies thus: "The District Committee pays the home £26 a-year for board, and 2s. 6d. for laundress. In one case, during an epidemic, two Jubilee nurses and mine were living one house, and the inclusive charge for board and lodging was 15s. per week, which I thought very fair. This does not include any kind of stimulant." Good housekeepers reckon a servant's board at £20 a year, when taken in conjunction with that of the family, and it is quite impossible to give any variety of suitable food under 10s. a week unless there be a number. We do not think it at all desirable that a nurse's board should be kept at the lowest possible sum necessary to sustain life. A nourishing diet, with a liberal amount of fruit, is necessary to maintain health. There is no economy in starving the steed that does the work, any more than there is in mismanaging the funds provided for food. There is a point beyond which even the best management cannot go, and to keep the nurses well is better han to save a shilling a week in their keep.

